# Automatic Piecewise Extreme Learning Machine-Based Model for *S*-Parameters of RF Power Amplifier

**DOI:** 10.3390/mi14040840

**Published:** 2023-04-13

**Authors:** Lulu Wang, Shaohua Zhou, Wenrao Fang, Wenhua Huang, Zhiqiang Yang, Chao Fu, Changkun Liu

**Affiliations:** 1 School of Micro-Nano Electronics, Zhejiang University, Hangzhou 310058, China; 2 Key Laboratory of Advanced Science and Technology on High Power Microwave, Northwest Institute of Nuclear Technology, Xi’an 710024, China; 3 Qingdao Institute for Marine Technology of Tianjin University, Qingdao 266200, China; 4 Research Center for Intelligent Chips and Devices, Zhejiang Lab, Hangzhou 311121, China; 5 School of Microelectronics, Tianjin University, Tianjin 300072, China

**Keywords:** power amplifier, auto-PW ELM, *S*-parameters, modeling, CMOS

## Abstract

This paper presents an automatic piecewise (Auto-PW) extreme learning machine (ELM) method for *S*-parameters modeling radio-frequency (RF) power amplifiers (PAs). A strategy based on splitting regions at the changing points of concave-convex characteristics is proposed, where each region adopts a piecewise ELM model. The verification is carried out with *S*-parameters measured on a 2.2–6.5 GHz complementary metal oxide semiconductor (CMOS) PA. Compared to the long-short term memory (LSTM), support vector regression (SVR), and conventional ELM modeling methods, the proposed method performs excellently. For example, the modeling speed is two orders of magnitude faster than SVR and LSTM, and the modeling accuracy is more than one order of magnitude higher than ELM.

## 1. Introduction

As a critical building block of wireless radio-frequency (RF) systems, the characteristics of power amplifiers (PAs) can directly affect the performance of wireless communication systems [1]. Therefore, it is an essential demand for RF circuit designers to design a well-behaved power amplifier for different application systems [2]. *S*-parameters are essential specifications to characterize microwave devices, which is no exception for power amplifiers [3]. The *S_11_* parameter reflects whether the input port is well-matched, the same as the *S_22_* parameter for the output port [2]. Generally, the power amplifier gain at the corresponding input power can be calculated with the *S_21_* parameter. In particular, the *S_12_* parameter can severely influence the circuit stability [2]. Above all, the *S*-parameters modeling of a power amplifier can realize the effective characterization and prediction of its performance.

Some researchers have performed *S*-parameters modeling in the literature. For example, in 2018, support vector regression (SVR) technique was adopted in *S*-parameters modeling by A. Khusro with modeling curves agreeing with the trend of the measured curves [4]. However, without mean square error (MSE) in the paper, obvious differences could be seen in the *S_12_* and *S_22_* parameters models. Furthermore, in 2019, A. Khusro et al. [5] used several improved artificial neural network (ANN) models for *S*-parameters modeling while models were only 10^−1^ orders of precision. It was worth noting that the whole models take at least the order of seconds. In 2020, *S*-parameters models of 1–10 GHz based on the piecewise SVR technique were constructed in Ref. [6] by M. Geng with MSE of 1.3 × 10^−2^ for *S_12_* parameters due to the strong nonlinear characteristic. Moreover, in 2021, M. Geng et al. adopted the long-short term memory (LSTM) and SVR methods in the measured 1–10 GHz *S*-parameters modeling [7]. Both models achieved an average accuracy of less than 10^−2^ orders of magnitude while the modeling time exceeded 10 s. In 2022, the support vector machine (SVM) method was used to model the measured *S*-parameters of 2.5–5.2 GHz CMOS PA varying with frequency at three different temperatures [8]. The model precision was only 10^−2^ orders of magnitude.

By summarizing the above literature, we can find much research on modeling *S*-parameters in the literature. However, they cannot show good modeling performance because of insufficient modeling precision or long modeling time. Particularly, modeling time becomes an important metric as testing and modeling requirements increase in mass production. In 2006, an extreme learning machine was proposed with the advantages of less adjustable parameters, better generalization performance, and fast convergence speed [9,10]. However, facing the large and strong nonlinear data samples, the model order increases as the model is complex, making the high precision demand challenging. Therefore, an improved ELM method based on model reduction technology is created to solve this problem.

This paper proposes an automatic piecewise ELM modeling method, which provides a segmentation strategy based on the change points of curve concavity and convexity as the segmentation points. After the segmentation, each segment is modeled with the ELM method, respectively. In this way, the aim of improving the precision of the model is realized with model order reduction. To verify the validity of the proposed method, an *S*-parameters measurement based on a 2.2–6.5 GHz CMOS PA is carried out. The modeling results show that the proposed model performs excellently compared with the conventional ELM, LSTM, and SVR methods, which achieves MSE below 5 × 10^−3^ and model time on the order of 10^−2^. Furthermore, the method presented in this paper is an excellent candidate for microwave device modeling.

## 2. Automatic Piecewise ELM Model

As can be seen from the above research literature, the data samples to be modeled are large and strongly nonlinear. Modeling and analyzing complex nonlinear data often yield time and storage consumption and sometimes increase uncertainty [11]. To accurately capture these parameters, piecewise methods are proposed to divide the solution state space into multiple segments [12]. Piecewise models can conveniently model with distinct nonlinear characteristics and have been proposed in much of the literature [12]. As for the conventional ELM method [13,14], the global dependence on local effects may also exist, making it perform poorly in modeling strong nonlinear characteristics [15,16]. Overall, an automatic piecewise model based on the ELM method is considered.

The analysis of piecewise models consists of the following two main steps: segmentation and modeling. Considering the segmentation strategy, there are many approaches to realize it. For example, it can be achieved based on the parameter extraction process in actual experiments [17,18] or according to the curve trend of the parameters [7,19]. Otherwise, dynamic programming is also commonly applied to find the optimal solution for segmentation [20]. This paper proposes a segmentation strategy based on the changing points of concave-convex parameter characteristics. The number of segments is determined by the curvature of piecewise curves, which means points where slight variation occurs of concave-convex traits, will not be considered splitting points.

Generally, nonlinear parameters can be fitted by functions containing multiple concave-convex characteristic fragments. Consider the nonlinear parameters as a continuous function φ(x) with *x* = (*x*_1_, *x*_2_, …, *x_i_*, …, *x_n_*), where $x_{i}\in R$. The function φ(x) is said to be concave with the following equation [21]:

(1) $$\varphi \left(cx_{i}+\left(1-c\right)x_{i+1}\right)\geq c\varphi \left(x_{i}\right)+\left(1-c\right)\varphi \left(x_{i+1}\right)$$

where c ∈[0,1], and $x_{1}<x_{2}$. In the same way, the function φ(x) is said to be convex with expression as follows:

(2) $$\varphi \left(cx_{i}+\left(1-c\right)x_{i+1}\right)\leq c\varphi \left(x_{i}\right)+\left(1-c\right)\varphi \left(x_{i+1}\right)$$

For the concave function, the derivative is monotonically decreasing about *x*, while for the convex function is the opposite. Furthermore, the concave function’s second derivative is negative, whereas the convex function is positive. For ∀i∈{1,2,…,n}, the changing points$\;x_{i}$ can be deduced with formulation as follows:

(3) $$\varphi^{\prime \prime }\left(x_{i}\right)*\varphi^{\prime \prime }\left(x_{i+1}\right)\leq 0\;$$

The second derivative represents the curvature of the function, which means the degree of deviation from the linear function. To avoid splitting regions at the points where data perturbation occurs, no action will be taken in the automatic splitting algorithm with the conditional statement as follows:

(4) $$\mathrm{if}\;\varphi^{\prime \prime }\left(x_{i}\right)<c_{0}$$

where threshold $c_{0}$ is a constant and is determined by the degree of data jittering. Moreover, to save time, the second derivative of the discrete measured data can be calculated directly by the discrete differential equation without fitting functions.

**Figure 1:**
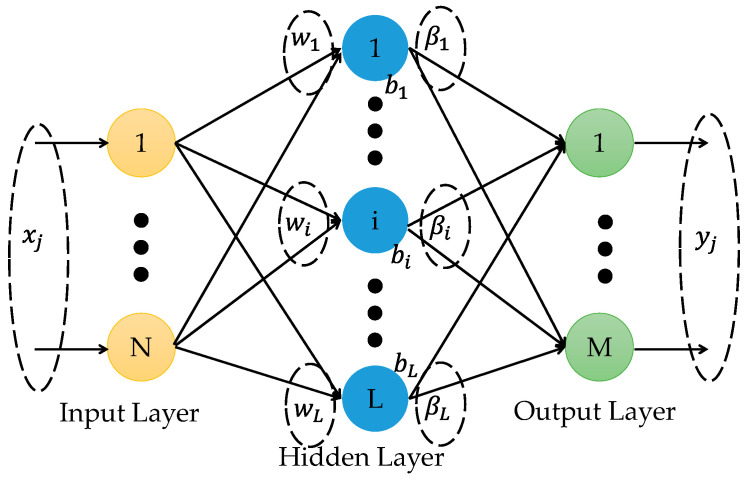
Basic ELM network architecture.

After the segmentation process of parameters, the piecewise models chosen are in demand. Due to the simple structure and impressive performance, the ELM model is a good choice for piecewise region modeling in this paper. In this section, a brief review of related research on ELM is introduced as follows. Extreme Learning Machine is a feed-forward neural network algorithm with a single hidden layer, as shown in Figure 1.

**Figure 2:**
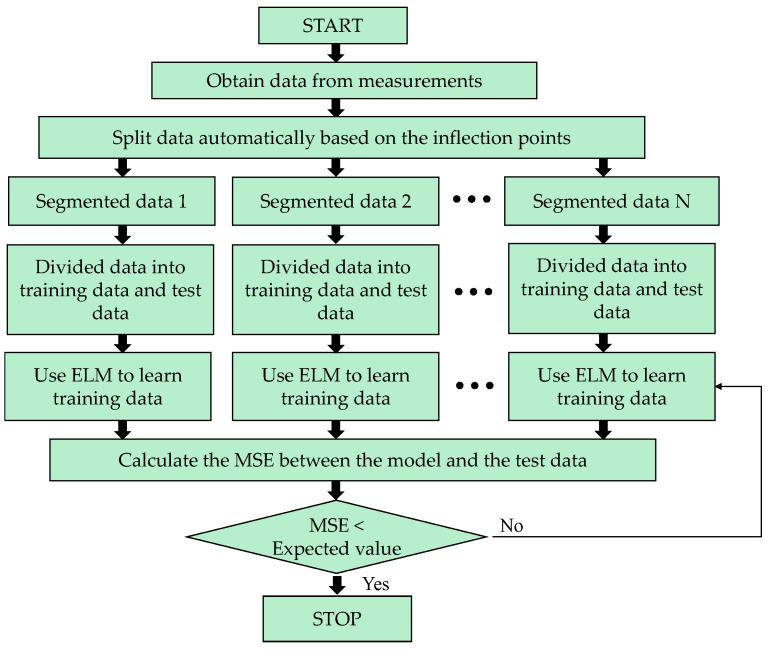
Flow chart of auto-PW ELM method.

Considering *T* arbitrary distinct samples ${\left\{\left(x_{j},y_{j}\right)\right\}}_{j=1}^{T}$, where $x_{j}\in R^{N}$ is the input vector, $y_{j}\in R^{M}\;$is the target response, and *L* denotes the number of hidden nodes set artificially. The weights and biases of the input and hidden layers ${\left\{\left(w_{i},b_{i}\right)\right\}}_{i=1}^{L}$ are generated randomly, where $w_{i}\in R^{N}$, $b_{i}\in R$. They remain unaltered after initiation, whereas the weight of the output layer ${\left\{\beta_{i}\right\}}_{i=1}^{L}$ is the only parameter the entire network needs to determine [22]. It has been proven that the single hidden layer forward network (SLFN) with random remote nodes has the universal approximation capability [9]. Thus, the ELM provides the best generalization performance at a breakneck learning speed. With the benefits mentioned above, ELM models are selected to apply in the piecewise regions. The flow chart of the auto-PW ELM method can be constructed in Figure 2.

## 3. Model Validation

**Figure 3:**
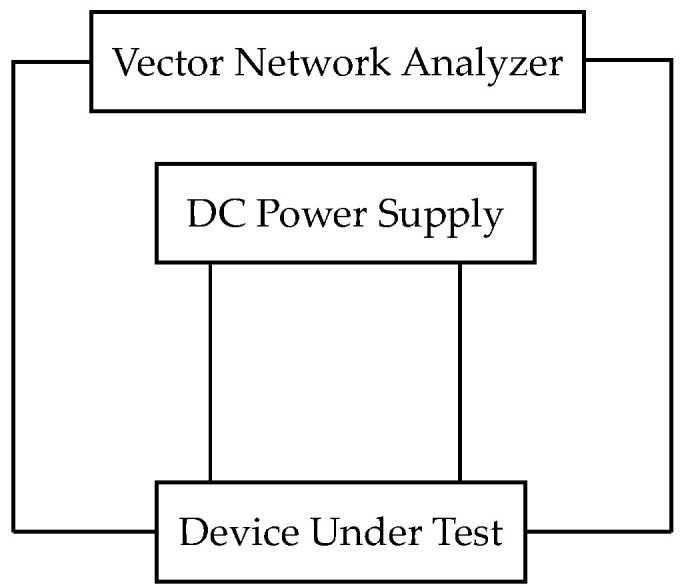
Measurement connections.

**Figure 4:**
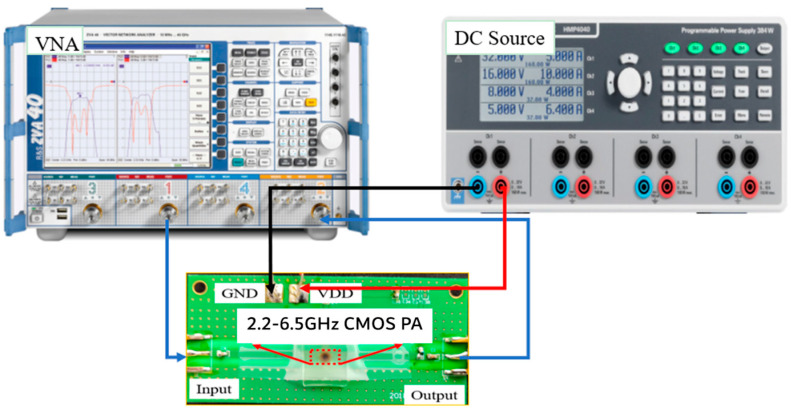
Schematic diagram of the experimental environment.

Model verification is carried out with *S*-parameters measured on a two-stage stacked CMOS PA with an input signal level of −20 dBm. The Class-A PAs structure was described in [23]. The connection schematic diagram of the measurement is shown in Figure 3, where DC bias is provided with a DC power supply and *S*-parameters measured by vector network analyzer (VNA). The VNA (ZVA40) and power supply (HMP4040) from R&S were used during the whole experiment. The measurement connection schematic of the experimental environment with instruments and DUT is given in Figure 4.

**Figure 5:**
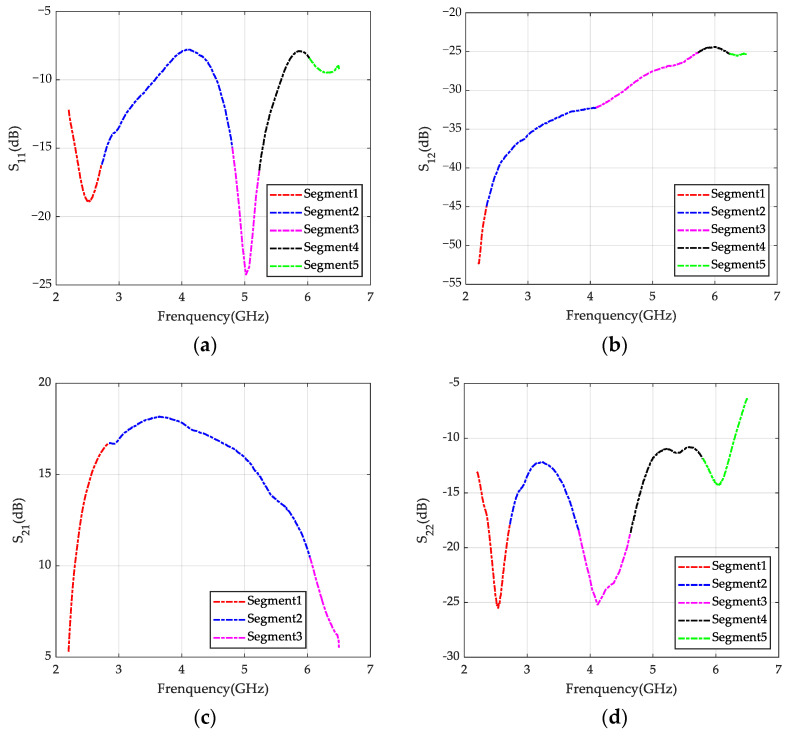
Results of splitting algorithm processing on *S*-parameters. (**a**) Piecewise curves of *S_11_* parameters. (**b**) Piecewise curves of *S_12_* parameters. (**c**) Piecewise curves of *S_21_* parameters. (**d**) Piecewise curves of *S_22_* parameters.

*S*-parameters with 801 sampling points in the 2.2–6.5 GHz frequency band are extracted within the measurements. The flow chart in Figure 2 shows that the segmentation strategy is adopted first to obtain the piecewise regions. Since the *S*-parameters (*S_11_*, *S_12_*, *S_21_*_,_ and *S_22_* parameters) show different variation trends with frequency, we split them into several areas separately. Especially the threshold $c_{0}$ value is set individually, counting on the different conditions. The results of splitting algorithm processing on *S*-parameters are depicted in Figure 5. Besides, the two adjacent regions are marked in other colors as apparent distinctions.

As Figure 5 illustrates, different *S*-parameters present distinctly different data distributions with frequency. The *S_11_* and *S_22_* parameters show strong nonlinearity with frequency variation, as the frequency band has multiple minimax points. Considering a combination of various concave and convex functions, *S_11_* and *S_22_* parameters can be divided into several regions according to our segmentation strategy. Besides, a conditional statement with a high value of the threshold $c_{0}$ is set to prevent splitting at slight data jitter, which may result in many segments. As for *S_12_* and *S_21_* parameters with nearly only one extreme point or none, less dramatic fluctuations in the curve are still detected under the proposed segmentation strategy. A more reasonable segmentation can be achieved based on an appropriate threshold$\;c_{0}$.

After the splitting algorithm processing, models (SVR, LSTM, ELM, and auto-PW ELM models) are adopted in *S*-parameters for verification. For consistency, parameters are divided, with half for training and half for testing in the same way. For fairness, the sigmoidal additive function is used as the activation function in conventional ELM and PW-ELM. Modeling results and discussion are as follows.

### 3.1. Modeling Results of S_11_ Parameters

**Figure 6:**
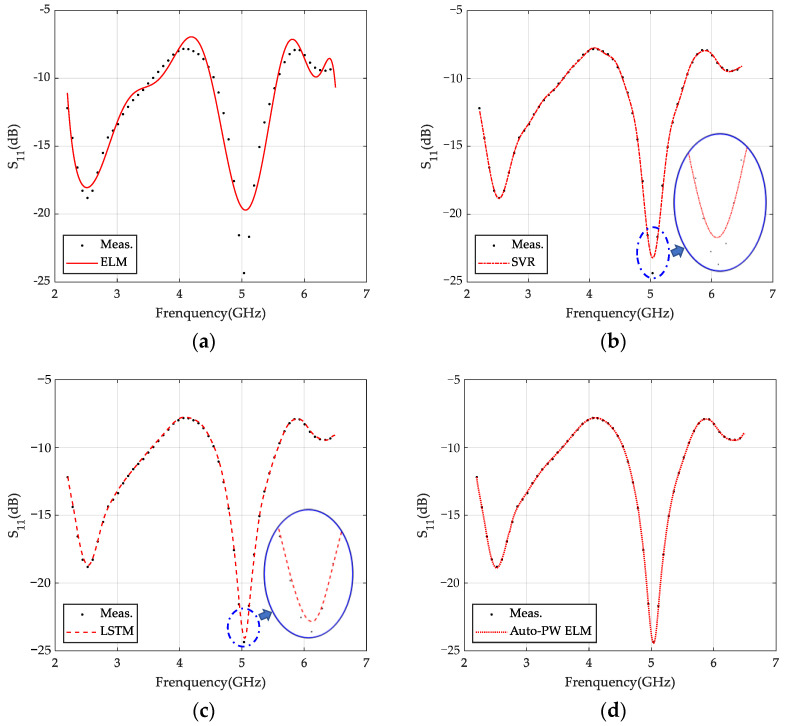
Modeling results of *S_11_* parameters. (**a**) Comparison of ELM model and measured data. (**b**) Comparison of SVR model and estimated data. (**c**) Comparison of LSTM model and measured data. (**d**) Comparison of automatic piecewise ELM model and measured data.

Figure 6 gives the modeling behavior of the *S_11_* parameters based on SVR, LSTM, ELM, and piecewise ELM models. As Figure 6a shows, the deviation between the ELM model and the measured data is significantly significant, especially at the beginning and end of the measured curve, indicating that the ELM model is not good at modeling strong nonlinear characteristics. Figure 6b,c shows similar modeling performances based on the SVR and LSTM models. The modeling curves coincide well with the changing trend of the measurement curve, except for the data in the peak region with zooms shown in blue circles. While considering the performance of the auto-PW ELM model shown in Figure 6d, good agreement of the model curve and measured curve can be achieved wherever in the beginning, estimated turn, or peak region.

### 3.2. Modeling Results of S_12_ Parameters

**Figure 7:**
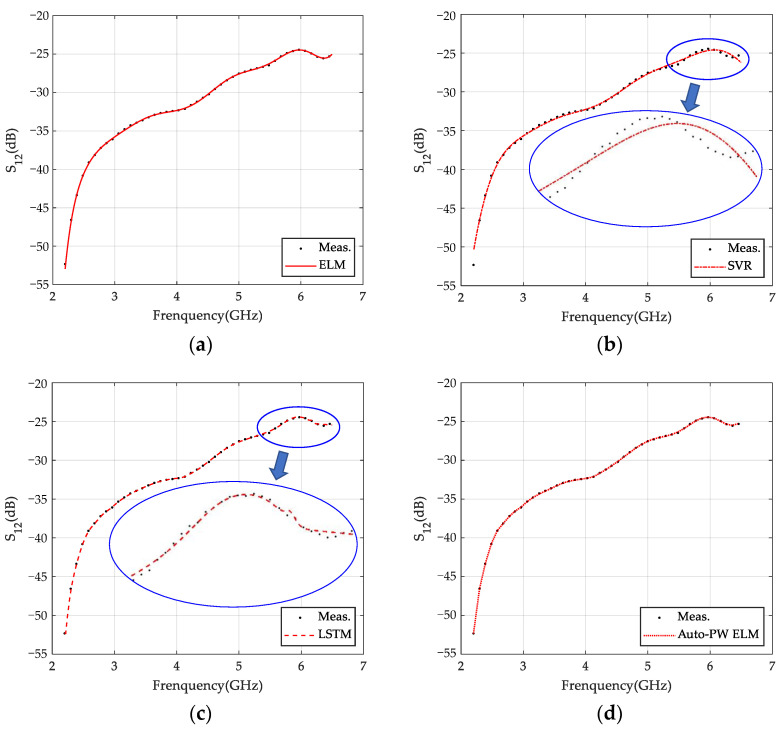
Modeling results of *S_12_* parameters. (**a**) Comparison of ELM model and measured data. (**b**) Comparison of SVR model and estimated data. (**c**) Comparison of LSTM model and measured data. (**d**) Comparison of automatic piecewise ELM model and measured data.

Figure 7 gives the modeling behavior of the *S_12_* parameters based on SVR, LSTM, ELM, and piecewise ELM models. As Figure 7 illustrates, the whole modeling curves within different methods coincide well with the changing trend of the measured curve. However, subtle fitting differences can be seen in the jitter of the measured angle. It is worth mentioning that the ELM model shows a decent performance of *S_12_* parameters modeling, which indicates that it is a good option for PA behavior modeling with weak nonlinearity characteristics. Furthermore, the proposed model agrees better with the measured curve than the other three.

### 3.3. Modeling Results of S_21_ Parameters

**Figure 8:**
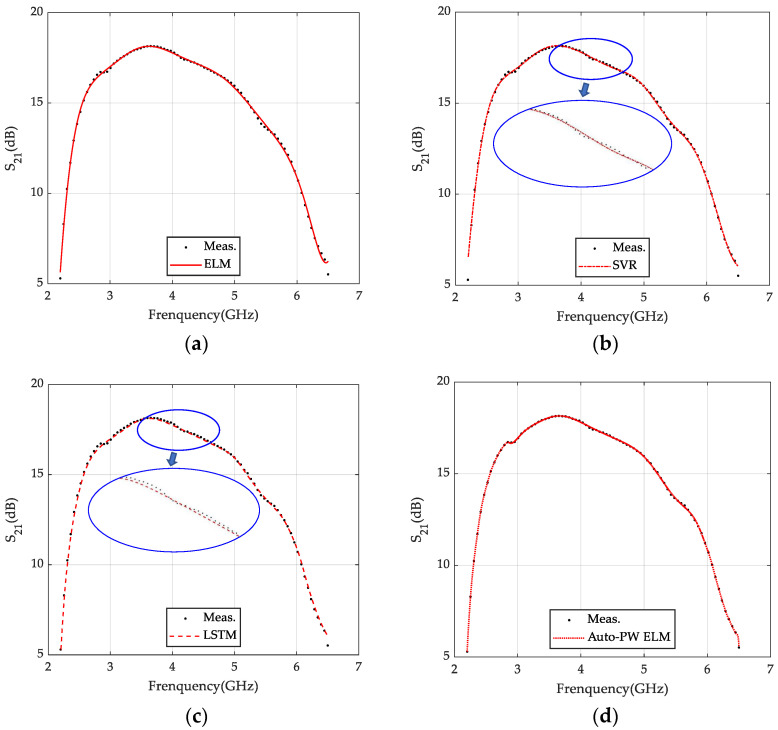
Modeling results of *S_21_* parameters. (**a**) Comparison of ELM model and measured data. (**b**) Comparison of SVR model and estimated data. (**c**) Comparison of LSTM model and measured data. (**d**) Comparison of automatic piecewise ELM model and measured data.

Figure 8 gives the modeling behavior of the *S_21_* parameters based on SVR, LSTM, ELM, and piecewise ELM models. Regarding the *S_21_* parameters, the four models perform a good fitting trend due to the slight fluctuation of the measured data. However, differences can be found in areas where convexity varied a lot. Overall, the proposed model offers superior modeling capabilities among the four models due to the reduced nonlinear strength of the piecewise method.

### 3.4. Modeling Results of S_22_ Parameters

**Figure 9:**
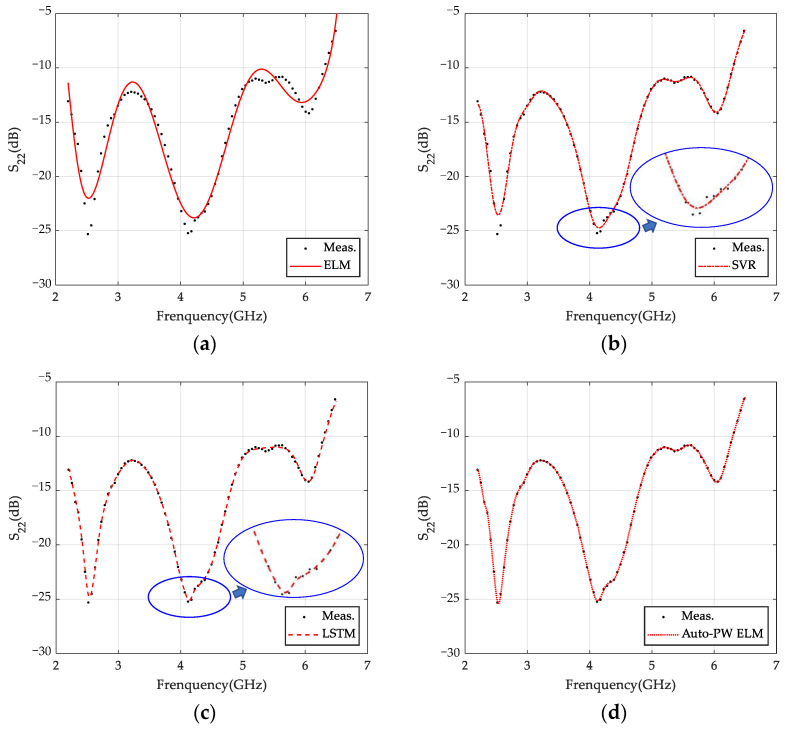
Modeling results of *S_22_* parameters. (**a**) Comparison of ELM model and measured data. (**b**) Comparison of SVR model and estimated data. (**c**) Comparison of LSTM model and measured data. (**d**) Comparison of automatic piecewise ELM model and measured data.

As the *S_22_* parameters modeling performance is based on SVR, LSTM, ELM, and piecewise ELM models displayed in Figure 9, a comparison of modeling performance can be observed. *S_22_* parameters show vital nonlinear characteristics as *S_11_* parameters, so as the modeling performance of the four different models. The conventional ELM model offers a significant deviation from the measured data, as expected in Figure 9a. While the SVR model and LSTM model exhibit a well-performed modeling ability, as Figure 9b,c illustrates. Moreover, the model accuracy of the two models is one magnitude higher than the ELM model. Figure 9d shows that the proposed model offers the most excellent performance compared with the other three models.

The modeling accuracy of different methods has been gathered in Table 1. The mean square error is selected as the performance evaluation indicator. For completeness, the execution time of different models is also provided. Different from the other three models, time spent for segmentation is also counted in Table 1 for auto-PW ELM models, with an average value of 0.03 s.

As can be observed, the modeling accuracy of the conventional ELM method falls significantly behind the auto-PW ELM method, especially for *S_11_* and *S_22_* parameters modeling, due to the strong nonlinearity. While it must be said that ELM performs relatively well in *S_12_* and *S_21_* parameters modeling with high speed and MSE below 0.02. In addition, from a time perspective, the ELM model shows a surprising speed in model training and can potentially be improved for the behavioral model. As for SVR and LSTM models, a similar modeling accuracy is obtained in Table 1, with a distribution of 6.3 × 10^−3^ to 8.1 × 10^−2^. At the same time, the execution time with an average value of 50 s seems to be a little large, almost 10^3^ times that of the ELM method. Significantly, the auto-PW ELM model achieves the best performance among the whole models with MSE under 5 × 10^−3^ and execution time below 10^−1^. Furthermore, the model accuracy based on auto-PW ELM is one or even three orders of magnitude higher than the other three models.

In addition, Table 2 summarizes the number of hidden neurons in terms of LSTM, ELM, and Auto-PW ELM models, which is an evaluation index of model complexity. Benefiting from the model order reduction technique, the automatic piecewise model provides each segment’s most minor hidden neurons. By the way, the SVR model does not involve the number of hidden neurons.

To the best of our understanding, the overall improvement in the modeling performance results from better modeling of the nonlinear characteristics in segments. In addition, it can be stated that the auto-PW method stands as a flexible and accurate model for *S*-parameters modeling.

## 4. Conclusions

This paper proposes an improved automatic piecewise model based on the ELM algorithm for *S*-parameters modeling of RF PAs. First, the model order reduction technique based on piecewise models is adopted to ensure the accurate modeling of strong nonlinear characteristics. Furthermore, ELM models with the significant benefit of high speed are adopted in the piecewise regions. Then, the segmentation strategy is proposed based on the changing points of concave-convex characteristics of parameters. A threshold for judging the degree of concave-convex characteristics is considered in the splitting algorithm, which enhances the algorithm’s capacity to suit different modeling parameters. Finally, measurements of *S*-parameters on the CMOS PA are carried out for model verification. Moreover, four different SVR, LSTM, ELM, and auto-PW ELM models are constructed with accuracy and execution time calculated. Based on the modeling results, the auto-PW model offers superior accuracy and speed capabilities with MSE under 5 × 10^−3^ and execution time below 10^−1^ s. The auto-PW model proposed in this paper is expected to achieve good results in modeling other RF/microwave devices or circuits.

## Figures and Tables

**Table 1:** Modeling accuracy of different models in terms of SVR, LSTM, ELM, and Auto-PW ELM.

	SVR		LSTM		ELM		Auto-PW ELM in Paper	
	MSE	Time/s	MSE	Time/s	MSE	Time/s	MSE	Time/s
*S_11_*	3.0 × 10^−2^	82	1.5 × 10^−2^	33	1.2	3.4 × 10^−2^	1.4 × 10^−3^	8.5 × 10^−2^
*S_12_*	8.1 × 10^−2^	55	9.2 × 10^−3^	52	1.2 × 10^−2^	5.2 × 10^−2^	6.9 × 10^−4^	8.2 × 10^−2^
*S_21_*	6.3 × 10^−3^	72	9.3 × 10^−3^	66	1.3 × 10^−2^	4.3 × 10^−2^	2.9 × 10^−3^	7.7 × 10^−2^
*S_22_*	8.5 × 10^−2^	77	3.1 × 10^−2^	53	3.2 × 10^−1^	4.4 × 10^−2^	4.3 × 10^−3^	9.5× 10^−2^

**Table 2:** Comparison of various approaches in terms of the number of hidden neurons.

Number of Hidden Neurons	LSTM	ELM	Auto-PW ELM in Paper
*S_11_*	80	80	5–15
*S_12_*	50	20	7–15
*S_21_*	50	20	5–15
*S_22_*	75	80	5–12

## Data Availability

Not applicable.
